# Supplementary material for: Adhering to Canada’s Food Guide Recommendations on Healthy Food Choices Increases the Daily Diet Cost: Insights from the PREDISE Study
Source: Nutrients. 2022 Sep 16;14(18):3818. doi: 10.3390/nu14183818 (PMC9502682; doi:10.3390/nu14183818)
Supplement: Supplementary file 1 [file nutrients-14-03818-s001.zip › nutrients-1863716-supplementary.pdf]

Table S1. Healthy Eating Food Index (HEFI)-2019 components, points and scoring system.[1]

	Component	Measurement	Maximum points	Unit	Standard for minimum score	Standard for maximum score
1	Vegetables and fruits	Total vegetables and fruits/ Total foods	20	RA/RA	No vegetables and no fruits	≥0.50
2	Whole grain foods	Total whole grain foods / Total foods	5	RA/RA	No whole grain foods	≥0.25
3	Grain foods ratio	Total whole grain foods / Total grain foods	5	RA/RA	No whole grain foods	1.0
4	Protein foods	Total protein foods / Total foods	5	RA/RA	No protein foods	≥0.25
5	Plant-based protein foods	Plant-based protein foods/ Total protein foods	5	RA/RA	No plant-based protein foods	>0.50
6	Beverages	(Plain water including carbonated + unsweetened beverages)/ Total beverages	10	g/g	No water and no unsweetened beverages	1.0
7	Fatty acids ratio	(Mono- + polyunsaturated fat)/ Total saturated fat	5	g/g	≤1.1	≥2.6
8	Saturated fats	Total saturated fat / energy	5	%E (kcal/kcal)	≥15%E	<10%E
9	Free sugars	Total free sugars / energy	10	%E (kcal/kcal)	≥20%E	<10%E
10	Sodium	Total sodium / energy	10	mg/kcal	≥2.0	<0.9
-	Total HEFI-2019	-	80	-	-	-

[1]Adapted from Brassard et al. 2022 (1)

Table S2. Methods used to calculate a price for the Bureau of Nutritional Science (BNS) food groups

Method used to compute a price	Bureau of Nutritional Science food groups
Prices obtained from the Nielsen database	01A- Pasta<br>01B-Rice<br>02A- White bread<br>03A- Whole wheat breads<br>03B- Other whole grain breads<br>04A- Rolls, bagels, pita bread, croutons, dumplings, matzo, tortilla<br>04B- Crackers and crispbreads<br>04C- Muffins and english muffins<br>04D- Pancakes and waffles<br>04E- Croissants, piecrusts & phyllo dough<br>05A- Whole grain, oats and high fiber breakfast cereals<br>06A- Breakfast cereal (other)<br>07A- Cookies, commercial<br>07C- Granola bar<br>08A- Pies, commercial<br>08C- Danishes, doughnuts and other pastries, commercial<br>09A- Ice cream<br>09B- Ice milk<br>09C- Frozen yoghurt<br>10A- Milk, whole<br>10B- Milk, 2%<br>10C- Milk, 1%<br>10D- Milk, skim<br>10I- Other types of milk (whey, buttermilk)<br>10J- Plant-based beverage (soy, almond, coconut)<br>14B- Cheese, less than 10% b.f.<br>14C- Cheese, 10% b.f. to 25% b.f.<br>14D- Cheese, more than 25% b.f.<br>15- Yoghurts, unknown fat %<br>15A- Yoghurts, less than 2% b.f.<br>15B- Yoghurts, more than 2.1% b.f.<br>16A- Egg<br>22A- Beef, lean only<br>22B- Beef, lean + fat<br>22C- Beef, ground<br>23A- Veal, lean only<br>23B- Veal, lean + fat (incl ground veal)<br>25A- Pork, fresh, lean only<br>25B- Pork, fresh, lean + fat (incl ground pork)

	25C- Bacon<br>25D- Ham, cured, lean only<br>25E- Ham, cured, lean + fat<br>27- Poultry, unknown type<br>27A- Chicken, meat only<br>27B- Chicken, meat + skin<br>27C- Turkey, meat only<br>28A- Liver<br>28B- Liver pate<br>30A- Sausage<br>32A- Luncheon meat<br>33A- Nuts<br>33B- Seeds<br>33C- Peanut butter and other nut spreads<br>34A- Fish, less than 6% total fat<br>34B- Fish, superior or equal to 6% total fat<br>35A- Shellfish<br>36- Vegetables, unknown type<br>36A- Beans<br>36B- Brocoli<br>36C- Cabbage and kale<br>36D- Cauliflower<br>36E- Carrots<br>36F- Celery<br>36G- Corn<br>36H- Lettuces & leafy greens (spinach, mustard greens, etc.)<br>36I- Mushrooms<br>36J- Onion, green onions, leeks, garlic<br>36K- Peas and snow peas<br>36L- Peppers, red & green<br>36M- Squashes<br>36N- Tomatoes<br>36O- Juices, tomato & vegetable<br>36P- Other veg (cucumber, immature beans, brussel sprouts, beets, turnips)<br>37A- Legume<br>37B- Foods made with vegetable proteins (tofu)<br>38A- Potato chips<br>38B- Fried or roasted potatoes<br>39A- Potato<br>40A- Citrus fruits (oranges, grapefruits, lemons, etc.)<br>40B- Apple<br>40C- Banana<br>40D- Cherries

	40E- Grapes and raisins<br>40F- Melons (canteloup, honeydew, watermelon)<br>40G- Peaches, nectarines<br>40H- Pears<br>40I- Pineapple<br>40J- Plums and prunes<br>40K- Strawberries<br>40L- Other fruits (blueberries, dates, kiwis, fruit salads, etc.)<br>42A- Popcorn, plain & pretzels<br>42B- Salty and high-fat snacks (incl tortilla chips)<br>43A- Candies, gums, etc.<br>43B- Ice pop, sherbert<br>44A- Chocolate bar<br>45A- Fruit juice<br>46A- Soft drinks - regular<br>46B- Soft drinks – diet<br>46C- Fruit drinks<br>46D- Other beverages (malted milk, chocolate beverage)<br>46E- Energy drink<br>46F- Vitamin water<br>50A- Soups with vegetables<br>50B- Soups without vegetables<br>50C- Gravies<br>54A- Energy bar<br>54B- Protein bar and shake<br>140C- Other breads, crackers, rolls, dumplings, bannocks, bagels, english muffins<br>140E- Pancakes & waffles<br>150A- Cakes, cheesecakes, shortcakes and brownies<br>150B- Cookie<br>150C- Danishes, turnovers & pastries<br>150D- Donuts<br>150E- Muffins<br>150F- Pies (including pie shell)<br>150H- Sweet rolls and breads<br>202B- Ice milk (recipe sub-group)<br>203A- Yoghurt (recipe sub-group)<br>204A- Cheese (recipe sub-group)<br>210- Meat dishes, unknown type<br>211A- Chicken (recipe sub-group)<br>213A- Sausage, with cured and luncheon meat (recipe sub-group)<br>215A- Chinese (recipe sub-group)

	216A- Mexican dish<br>217A- Legume dishes without meat<br>219F- Hotdogs<br>220B- French fries and hash brown potatoes<br>220F- Vegetables (mixed with other stuff)<br>226A- Nuts and seeds (recipe sub-group)<br>229A- Sweet snacks, sugar, candies (recipe sub-group)<br>230B- Soup, dehydrated<br>231D- Milk-based beverage (milk shakes, malted milk, hot cocoa, instant breakfast, etc.)<br>2223- Beef and veal, lean + fat
Prices obtained using statistical data from the Government of Canada (2)	01C-Cereal grains and flours;<br>10F-Milk, evaporated, 2%;<br>10G-Milk, evaporated, skim;<br>17A-Butter;<br>18A-Regular tub margarine;<br>18B-Calorie-reduced tub margarine;<br>21A-Vegetable oils;<br>41A-Sugars (white and brown);<br>50D-Sauces (white, bearnaise, soya, tartar, ketchup, etc.);<br>51B-Coffee;
Prices obtained using statistical data from the Government of Quebec (3)	41C-Other sugars (syrups, molasses, honey, etc);
Prices of similar foods compared using a supermarket website. Then the missing price was replaced with the price of a comparable food from the Nielsen database, Statistics Canada database, or the Institut de la statistique du Québec database with the application of a factor if necessary	10K-Goat and sheep milk;<br>13A-Whipping cream;<br>13B-Table cream;<br>13C- Half & half cream;<br>13D-Sour cream;<br>14A-Cottage cheese;<br>21B-Animal fats;<br>21C-Shortening;<br>24A-Lamb, lean only;<br>24B-Lamb, lean + fat (incl ground lamb);<br>25C-Bacon;<br>27E-Other birds (duck, pheasant, pigeon);<br>29A-Offal;<br>41B-Jams, jellies and marmalade;<br>41D-Sugar substitutes;

	43C-Gelatin, dessert toppings and pudding mixes, commercial;<br>46C-Fruit drinks;<br>46G-Sports drink;<br>50E-Salad dressings (with or without oil);<br>54C-Meal replacements;<br>130F-Rice (recipe sub-group);<br>205A-Milk dessert (recipe sub-group);<br>212A-Fish, with less than 6% fat before cooking (recipe sub-group);<br>212C-Shellfish (recipe sub-group);<br>231J-Protein powder;
Prices obtained from the 2016 annual report of the Société des alcools du Québec (4)	47A-Spirits;<br>47B-Liqueurs;<br>48A-Wine;<br>49A-Beer;<br>231G-Alcoholic beverages;
No price computed	31A-Game meat;<br>50F-Seasonings (salt, vinegar, etc.);<br>51C-Water (well & mineral);<br>52A-Babyfood product;<br>53A-Spices;<br>53B-Others (baking soda, baking powder, yeast, etc.);

T**able S3.** Non-energy adjusted daily diet cost according to sociodemographic characteristics[1].

	Diet cost<br>$CAD/day (95%CI)[2]
**All**	12.05 (11.80, 12.30)
**Sex**	
Women	10.66 (10.37, 10.96)
Men	13.45 (13.04, 13.86)
**Age**	
18-34 y	11.75 (11.30, 12.21)
35-49 y	12.18 (11.71, 12.65)
50-65 y	12.25 (11.87, 12.63)
**Education[3]**	
None, high school or trade diploma	11.84 (11.25, 12.43)
CEGEP	11.98 (11.51, 12.45)
University	12.21 (11.85, 12.57)
**Household income[3]**	
< 30,000 $CAD	10.58 (9.88, 11.27)
30,000 to < 60,000 $CAD	11.95 (11.43, 12.46)
60,000 to < 90,000 $CAD	12.09 (11.59, 12.60)
≥ 90,000 $CAD	12.86 (12.45, 13.27)
**Administrative region**	
Capitale-Nationale/Chaudière-Appalaches	12.41 (11.97, 12.85)
Estrie	11.83 (11.20, 12.46)
Mauricie	12.32 (11.41, 13.23)
Montreal	11.58 (11.19, 11.98)
Saguenay-Lac St-Jean	12.30 (11.45, 13.15)

[1] CAD, Canadian dollars; CEGEP, Collège d'Enseignement Général et Professionnel;
[2] Because of their descriptive nature, these mean costs are not based on the National Cancer Institute's multivariate method.
[3] Missing sociodemographic characteristics have been imputed. See the Methods section for details.

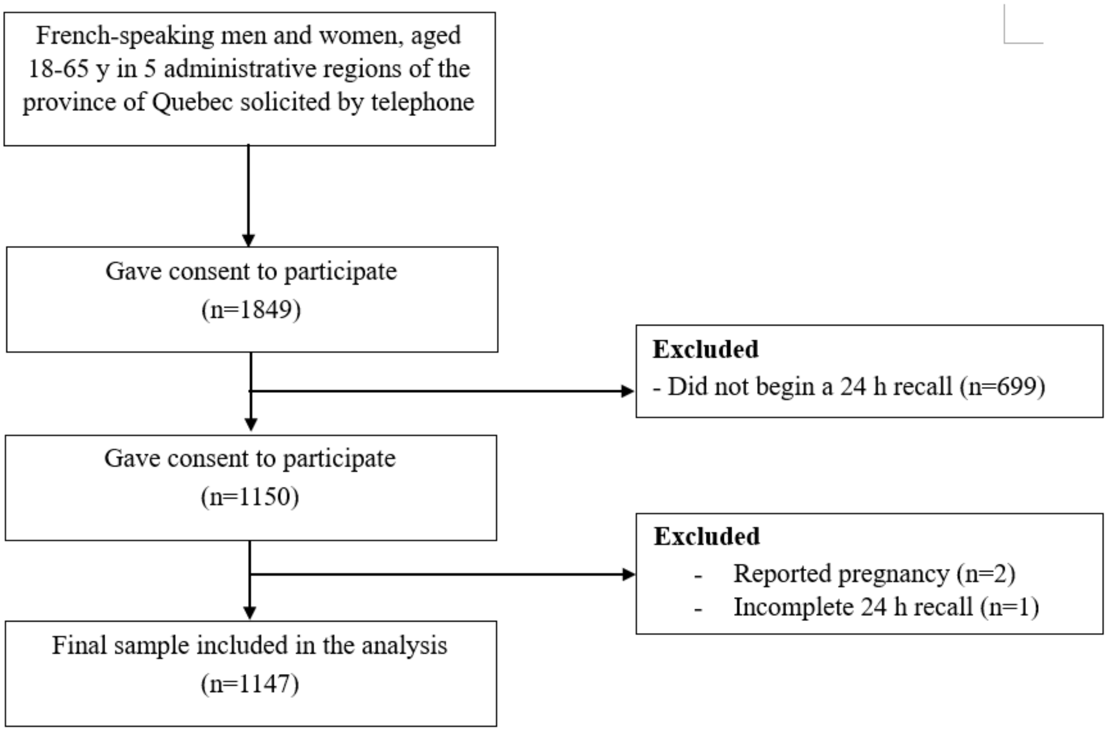

Figure S1. Flow chart of the Predicteurs Individuels, Sociaux et Environnementaux (PREDISE) study participants included in the analytical sample.

References

1. Brassard, D.; Elvidge Munene, L.-A.; St Pierre, S.; Gonzalez, A.; Guenther, P.M.; Jessri, M.; Vena, J.E.; Olstad, D.L.; Vatanparast, H.; Prowse, R.; et al. Evaluation of the Healthy Eating Food Index (HEFI)-2019 Measuring Adherence to Canada's Food Guide 2019 Recommendations on Healthy Food Choices. *Appl. Physiol. Nutr. Metab.* **2022**, *47*, 582–594. https://doi.org/10.1139/apnm-2021-0416.
2. Gouvernement du Canada, S.C. Prix de Détail Moyens Mensuels Pour les Aliments et Autres Produits Sélectionnés. Available online: https://www150.statcan.gc.ca/t1/tbl1/fr/tv.action?pid=1810000201 (accessed on 18 January 2022).
3. Québec, I. de la statistique du Répartition du Prix Moyen de Vente du Miel Selon le Marché, par Regroupement de Régions Administratives, Québec. Available online: https://statistique.quebec.ca/fr/produit/tableau/repartition-du-prix-moyen-de-vente-du-miel-selon-le-marche-par-regroupement-de-regions-administratives-quebec (accessed on 18 January 2022).
4. SAQ. Rapport Annuel 2016. 2016. Available online: https://saqblobmktg.blob.core.windows.net/documents/rapport-annuel-2015-16-fr.pdf (accessed on 18 January 2022).
